# The presence of experienced individuals enhance the behavior and survival of reintroduced woolly monkeys in Colombia

**DOI:** 10.1007/s10329-024-01156-2

**Published:** 2024-10-25

**Authors:** Mariana Gómez-Muñoz, Mónica A. Ramírez, Jairo Pérez-Torres, Pablo R. Stevenson

**Affiliations:** 1 Facultad de Estudios Ambientales y Rurales, Pontificia Universidad Javeriana, Bogotá, Colombia; 2 Laboratorio de Ecología de Bosques Tropicales y Primatología (LEBTYP), Departamento de Ciencias Biológicas, Universidad de Los Andes, Bogotá, Colombia; 3 Laboratorio de Ecología Funcional (LEF), Departamento de Biología, Facultad de Ciencias, Unidad de Ecología y Sistemática (UNESIS), Pontificia Universidad Javeriana, Bogotá, Colombia

**Keywords:** Behavioral adjustment, Diet, *Lagothrix lagothricha*, Primate reintroduction, Social learning, Use of space

## Abstract

Primate populations are under threat due to human activities, like illegal trafficking, requiring conservation efforts such as reintroduction programs. However, these initiatives often encounter challenges, such as aspects of individual behavior related to movement and foraging. The presence of experienced conspecifics has been suggested to improve the success of these programs. This study focuses on woolly monkeys and examines how the presence of experienced conspecifics influences the performance of reintroduced individuals. Focal animal sampling was used to collect data on proximity, diet composition, home range, and use of vertical strata of three groups of reintroduced woolly monkeys. Data was analyzed for the first 2 and 6 months after release of individuals. The results reveal that the involvement of experienced conspecifics speeds up the process of adaptation, particularly in terms of diet diversity and spatial utilization; however, differences in individual responses were also prevalent. Our findings highlight the importance of incorporating individuals with prior wild experience into reintroduction groups for improving the likelihood of success. Additionally, the study underscores the role of social learning in captive management practices, aiding in post-release behavioral adaptation and survival. This research offers valuable insights for primate conservation, emphasizing the significance of considering the presence of experienced individuals and possible social learning processes in the planning and execution of effective reintroduction efforts.

**Supplementary Information:**

The online version contains supplementary material available at 10.1007/s10329-024-01156-2.

## Introduction

Approximately, 66% of the primate species are threatened (Estrada et al. 2017), due to human activities such as disturbance of natural ecosystems and hunting. Given the conservation status of primate populations and their ecological importance (Peres and Palacios 2007; Peres and van Roosmalen 2009; Stevenson and Guzmán-Caro 2013), reintroduction programs have been recognized as significant conservation tools (IUCN 2013; Seddon et al. 2014). However, globally, such initiatives tend to have low success rates (Reading et al. 2013; Seddon et al. 2014) and are usually expensive (Kavanagh and Caldecott 2013; Kleiman 1989; Kleiman et al. 1991; Ramírez 2020). Thus, understanding the primary factors influencing reintroduction success is crucial.

Some studies have suggested that the presence of an experienced conspecific might help in the success of the reintroduction process (Pottie et al. 2021; Reading et al. 2013; Riedler et al. 2010; Stoinski et al. 2003). The presence of experienced individuals appears to facilitate a social learning process, aiding in the acclimatization of released individuals to the new environment. Social learning involves behavioral changes resulting from observations or interactions with group members (Galef 2006) and favors the maintenance of critical knowledge and skills within primate communities, such as survival abilities (Box 1991; Custance et al. 2002).

Movement and dispersal from the release site have been consistently reported as major challenges in reintroduction programs, significantly affecting their success rates (Armstrong and Seddon 2007; Berger-Tal et al. 2020; Stamps and Swaisgood 2007). This includes the assessment of home range and vertical strata utilization (Marines-Macías et al. 2018), as these are integral components of the behavioral adaptation process. In addition to movement challenges, individuals involved in reintroduction programs often need to familiarize themselves with the new environment, especially in terms of recognizing suitable foraging sites (Stoinski et al. 2003). This is particularly significant as choices regarding dietary composition can significantly influence the well-being of individuals (Rapaport and Brown 2008).

Woolly monkeys (*Lagothrix lagothricha)* are one of the largest primates in the Neotropics and play an important ecological role as seed dispersers (Peres and Palacios 2007; Stevenson 2002, 2011; Stevenson and Guzmán-Caro 2013). In the wild, woolly monkeys primarily inhabit canopy levels ranging from 12 to 18 m (Stevenson and Quiñones 1993), but occasionally descend to the understory (Defler 1999). There are two subspecies in Colombia, *Lagothrix lagothricha lagothricha* and *L. l. lugens* (Botero and Stevenson 2014). Both subspecies face conservation concerns as, according to the International Union for the Conservation of Nature (IUCN) (2021), they are considered as Vulnerable (VU) and as Critically Endangered (CR), respectively. This conservation status is due to human activities such as disturbance of natural ecosystems, hunting, and illegal trade. The management of individuals seized or confiscated from illegal trafficking poses a challenge due to the high number of individuals and limited infrastructure of facilities responsible for carrying out rehabilitation and reintroduction processes (Mendivelso and Montenegro 2007). Furthermore, legal gaps and poor interagency coordination hamper the effective enforcement of existing laws for the development of these programs (Bennet et al. 2013).

This study is part of a long-term project initiated in 2016, involving the rehabilitation and reintroduction of woolly monkeys rescued from wildlife trafficking in Colombia. Here, we analyze the data for three woolly monkey reintroduction events in Rey Zamuro–Matarredonda reserve (Meta, Colombia), where each group (A, B, and C) differed in the presence or absence of individuals that were previously released and had survived in the area (from now on “experienced conspecifics”). For instance, when Group A was released, there were no other individuals in the area. On the contrary, when Groups B and C were released, experienced conspecifics were present (1 and 2, respectively). This study aimed to investigate if the presence of experienced individuals could affect the behavioral adaptation of reintroduced woolly monkeys, possibly through social learning. We tested the hypothesis that their behavior is influenced by the presence of experienced conspecifics, in terms of (1) diet composition, (2) home range, and (3) vertical strata. If social knowledge and learning are important, we expected that (1) Groups B and C will diversify their diet more quickly, incorporating a wider range of wild fruits and natural sources than the first group (Group A). (2) Woolly monkeys in Groups B and C, with access to experienced conspecifics, will establish larger home ranges more rapidly than Group A. (3) Groups B and C will use higher vertical strata more rapidly than Group A. We assessed these predictions for the first 2 months and first 6 months (as the initial months are the critical period, with the highest loss rates). We also expected that at the end of the monitoring period, diet, home range, and use of vertical strata will be more similar among groups.

## Methods

### Study area

This study took place in the natural reserve Rey Zamuro–Matarredonda, located in San Martín in the Department of Meta, Colombia (N 03°32′44.8'' W 73°23′55.7''). It covers an area of 1552.5 ha (Ocampo-Peñuela and Etter 2013), of which approximately 40% corresponds to forest cover and 60% to savannas and grasslands (Casallas-Pabón et al. 2018; Díaz-Pulido et al. 2010). The rainfall varies from 2200 to 5100 mm in areas near the mountain range (Ministerio de Turismo 2010), with altitudes ranging from 260 to 300 m above sea level and mean temperature is 25.6 °C. Based on fruit traps and phenological transects, Ramírez (2020) estimated a fruit production of 1200 kg/ha.yr, which, according to Ramírez and Stevenson (2020), complies with the minimum amount of fruit production required to maintain populations of woolly monkeys (400 kg/ha.yr). There are five different primate species in the reserve: *Sapajus apella, Alouatta seniculus, Saimiri cassiquiarensis, Plecturocebus ornatus,* and *Aotus brumbacki.* There are not wild populations of woolly monkeys, although the older residents do remember these monkeys in the area.

### Study groups

This study covered three reintroduction events of woolly monkeys in the reserve. Group A, consisting of six individuals (adult females: Amalia, Micaela, Yara; adult males: Aurelio, Kaly, Loui), was released in November 2018. Following this, Group B, with three individuals (adult female: Lara, juvenile female: Lua, subadult male: Pistón), was released in November 2019. When the introduction of Group B occurred, Micaela, the only survivor of Group A, was present in the area. Furthermore, Group C was released in December 2022, including five individuals (adult male: Tyson; juvenile females: Camila, Inga; juvenile males: Botija, Otto), when Micaela from Group A and Lua from Group B were in the area.

Identifications of individuals were done using age and sex categories and physical characteristics such as body size, facial shapes, and genital markings. All the individuals were considered suitable for the reintroduction process, as they demonstrated behavioral patterns in captivity (before and during the rehabilitation) similar to wild populations reported by Stevenson (2006) (Suplementary information). Additionally, the limited available history of each individual can be found in Table 1 (for instance, the age when captured was unknown for all individuals). The sample size for each group fluctuated due to factors such as predation, falling from trees, or disappearance. Releases were done in the same season, allowing individuals to experience similar fruit productivity patterns, which are relatively stable for the region (Bautista 2019). Also, releases were done in the same location in the study area. For each group, only the data of individuals relevant to their respective release were included. Specifically, Micaela’s behavior was not included in Group B, and neither Micaela’s nor Lua’s were included in Group C. In 2018, Group A was released as a baseline or control group, with no other individuals present in the area. By contrast,, when Groups B and C were released, individuals from previous releases were already present in the area, providing a different social setting. This context offers valuable insights into how familiarity of previous released individuals with the environment might influence the behavioral adaptation of the newly reintroduced groups. Another difference between Group A and Groups B and C is that the latter two had short periods of in situ rehabilitation within the reserve, specifically 2 and 1 months each. During this period, individuals were located in an acclimatization enclosure, where we provided a food supply and monitored their behavior. Non-agonistic interactions between individuals in the acclimatization enclosure and free-ranging individuals were observed. These interactions seem to have incentivized free-ranging individuals to remain at the release site and stimulated the group cohesion, but it is unlikely that this had a direct effect on the future patterns of habitat use or diet.

**Table 1:** Individuals and corresponding groups (A, B, and C) of reintroduced woolly monkeys in Reserve Rey Zamuro–Matarredonda

Individual	Age–sex	Group	Reported time in captivity* (years)	Rehabilitation period (months)	Activity X^2^ ** (p)	Survival time (months)	Experienced individuals	Status at the end of the study
Kaly	Adult male	A	1	5	16.66 (< 0.01)	0	None	Disappeared
Loui	Subadult male	A	2	5	8.84 (0.01)	0.1	None	Death (tree falling)
Yara	Adult female	A	1.5	5	9.84 (0.01)	5	None	Predated (felid)
Amalia	Adult female	A	1	5	5.93 (0.05)	0.4	None	Predated (felid)
Aurelio	Adult male	A	1	5	2.44 (0.29)	1	None	Disappeared
Micaela	Adult female	A	1	5	12.09 (< 0.01)	6	None	Alive
Lara	Adult female	B	0.5	2	7.24 (0.02)	5	Micaela	Disappeared
Pistón	Subadult male	B	1	2	9.31 (< 0.01)	5	Micaela	Disappeared
Lua	Juvenile female	B	1	2	16.15 (0.01)	5	Micaela	Alive
Tyson	Adult male	C	0.8	5	9.03 (0.01)	0.1	Micaela, Lua	Death (tree falling)
Botija	Juvenile male	C	2.8	5	24.77 (< 0.01)	6	Micaela, Lua	Alive
Inga	Juvenile female	C	3.6	5	27.37 (< 0.01)	6	Micaela, Lua	Alive
Camila	Juvenile female	C	0.7	5	11.49 (< 0.01)	6	Micaela, Lua	Alive
Otto	Juvenile male	C	0.5	5	14.67 (< 0.01)	6	Micaela, Lua	Alive

^*^Captivity period in rescue center

^**^X^2^ comparing behavior patterns (including feeding, moving, and resting) between reintroduced individuals and wild woolly monkeys (X^2^ mean values in captive woolly monkeys in Colombia range from 5 to 55, Guzmán-Caro and Stevenson (2014), so we discarded all individuals with X^2^ > 30

### Food supply

Between 3 and 4 months after their release, elevated platforms (feeders) were used to provide all three groups with a food supply based on fruits and vegetables available in local markets and wild fruits found at the release site. For the different releases, feeders were placed in the same area and the same platforms were used for all the groups. Following Patiño (2019), the daily dietary supplement corresponded to 10–15% of the individual’s body weight. For all groups, the frequency of the supply was gradually reduced to stimulate the search for food.

### Focal sampling

Information on habitat use and consumed items was collected using focal animal sampling (Altmann 1974). During the 1st week after the release of each group, individual focal samplings of all released individuals were carried out between 06:00 h and 18:00 h. Later, for six consecutive months after the release, a minimum of three focal samplings per individual and per month were carried out (however, due to the COVID-19 pandemic, Group B was monitored for only 5 months after the release). Once the group was located, observers selected an individual as the focal subject. Focal samples were evenly distributed between individuals in the groups. If an individual was out of sight, data was recorded as OOS and, if the observer could not find the focal for the next 4 min, the focal follow was ended. This resulted in a total of 1092 h of observations (408 h for Group A, 312 for Group B, and 372 for Group C). For each group, two field researchers gathered the data systematically by following a standardized protocol (Stevenson 2006).

### Data collection

Instantaneous samples for the focal individual were taken every 10 min. For each sample, the activity classified as moving, resting, feeding, or social interactions (González and Stevenson 2010) and identity of individuals in less than 5 m from the focal were recorded. This method was implemented in the release site, as well as in the rehabilitation period, which varied in length for each group (Table 1). In addition, feeding bouts, defined as the events wherein the individual started manipulating fruits until it left the tree or stopped feeding for more than 1 min, were recorded ad libitum (Steveson 2004; Stevenson et al. 1994). For each feeding bout, diet composition was categorized into six food categories: feeder (platform), fruits, leaves, flowers, arthropods, and others (vertebrates and termite mound material) (Defler and Defler 1996; Di Fiore 2004). In this study, our focus was on fruit species, as woolly monkeys are primarily frugivorous, and their behavior is highly dependent from fruit abundance (Stevenson et al. 1994), as well as population densities (Stevenson 2014; Ramírez and Stevenson 2020). To identify fruit species, we collected samples that were later identified by P.R.S using herbarium material and guides (e.g., Stevenson et al. 2000a). During the study, 4144 feeding bouts were recorded (905 for Group A, 1140 for Group B, and 2099 for Group C). Group composition was recorded daily, including encounters with individuals of other primate species. Also, the location position was recorded as a waypoint with a handheld GPS device every 30 min. For the use of vertical strata, we visually estimated and recorded the height above the ground of the individual’s location (after a training period using laser equipment) at 30-min intervals. For each reintroduction event, data was collected following the same protocol to allow comparisons.

### Data analyses

Considering that learning can occur through proximity to experienced individuals, the percentage of samples in which focal individuals of Groups B and C were in proximity (< 5 m) to experienced individuals was estimated. Diet composition, for food types, was compared between Groups A, B, and C, using two non-metric multidimensional scaling (NMDS) based on a Bray–Curtis index (Bray and Curtis 1957). This analysis was employed to visually compare the diet of the different groups, where lower stress values indicate a better representation of the data (Clarke 1993). Additionally, an ANOSIM test was performed to statistically discriminate between groups. Rarefaction curves with their 95% confidence intervals were performed to identify differences in richness of consumed fruit species between groups (Burns 2009). Analyses were conducted for both the initial 2 months and the total monitoring period. Analyses were conducted using the ‘vegan’ package (Oksanen et al. 2022) in R version 4.1.1 (R Core Team 2021). Graphs were generated using the ggplot2 package (Wickham 2016) in R version 4.1.1 (R Core Team 2021).

For home range and core areas, waypoints were used to estimate home range sizes. Kernel density estimates at a 95% and 50% probability utilization distribution (UD) (Worton 1989) were calculated. Estimation of home range size was done using the package *adehabitatHR* of R (Calenge 2006; R Core Team 2021) and QGIS (version 3.26.3). Additionally, a grid (100 m × 100 m) was built to assess the frequency of visits to each quadrant by each group (to calculate a rarefaction curve to represent the cumulative number of hectares as a function of sampling time). For vertical strata, a Kruskall–Wallis tests was performed to establish differences in the use of vertical strata between groups across the first 2 months after the release and for the total monitoring period. Then, a Dunn’s post hoc test was applied. Analyses were done using the package *stats* of R (R Core Team 2021). Graphs were generated using the ggplot2 package (Wickham 2016) in R version 4.1.1 (R Core Team 2021).

## Results

### Proximity

During the first 2 months, individuals form Group B were in proximity (< 5 m) with an experienced conspecific in 8% of the samples, and this increased slightly to 9% for the total monitoring period. Conversely, individuals from Group C were in proximity (< 5 m) with an experienced conspecific in 25% of the samples during the first 2 months, which decreased to 15% for the entire monitoring period. Additionally, there were differences in the rates of proximity among individuals of the same group (Table 2).

**Table 2:** Percentage of samples in which focal individuals were in proximity (< 5 m) to experienced individuals in Reserve Rey Zamuro–Matarredonda

Individual	Group	Number of experienced individuals present	Percentage		N*
			2 months	6 months	
Lara	B	1	2	4	360
Lua	B	1	16	12	943
Pistón	B	1	5	10	832
Botija	C	2	36	24	761
Otto	C	2	32	17	387
Inga	C	2	16	12	624
Camila	C	2	18	8	428

^*^N total number of scan samples

### Diet composition

**Fig. 1:**
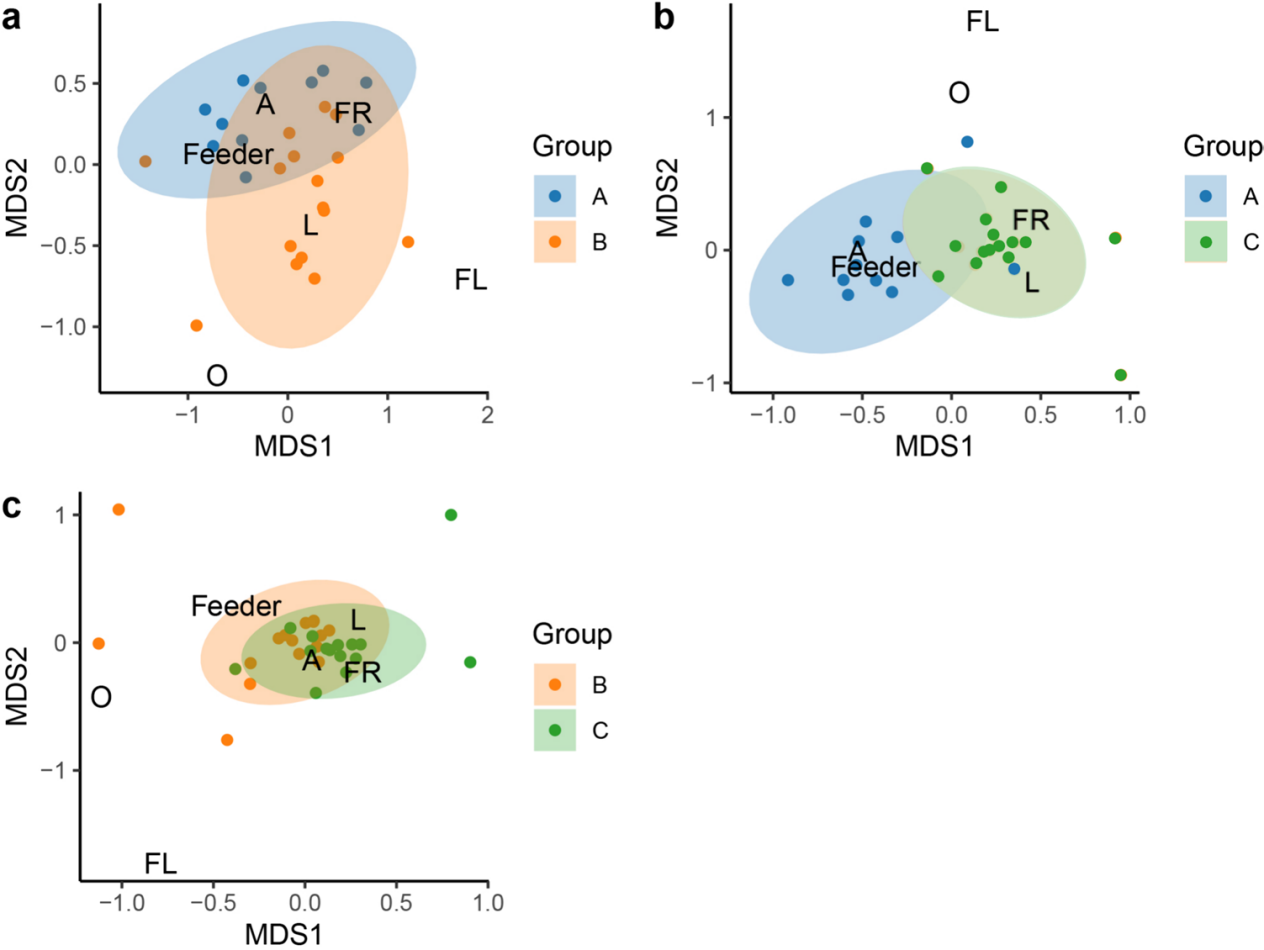
Diet composition variation among reintroduced woolly monkeys (Groups A, B, and C) at Reserve Rey Zamuro–Matarredonda. Non-metric multidimensional scaling ordination (NMDS) of diet composition during the initial 2- month period of a) Groups A and B, **b** A and C, and **c** B and C (the only not significant comparison). The diet components considered include arthropods (A), fruits (FR), flowers (FL), feeders, leaves (L), and others (O). In the plot, different groups (A, B and C) are distinguished by color (Group A by blue, Group B by orange, and Group C by green)

For the first 2 months, diet composition obtained from NMDS showed differences between Groups A and B (stress = 0.136) (Fig. 1a), between A and C (stress = 0.099) (Fig. 1b), and between B and C (stress = 0.111) (Fig. 1c). The ANOSIM analysis showed dissimilarity in diet composition between Groups A and B (R = 0.32, *p* = 0.01), between A and C (R = 0.54, *p* = 0.01), and between B and C (R = 0.12, *p* = 0.005). In contrast, for the 6-month period, the diet compositions of Groups A and B and Groups A and C were less dissimilar and there were no significant differences in the diet composition between Groups B and C (Supplementary information).

**Fig. 2:**
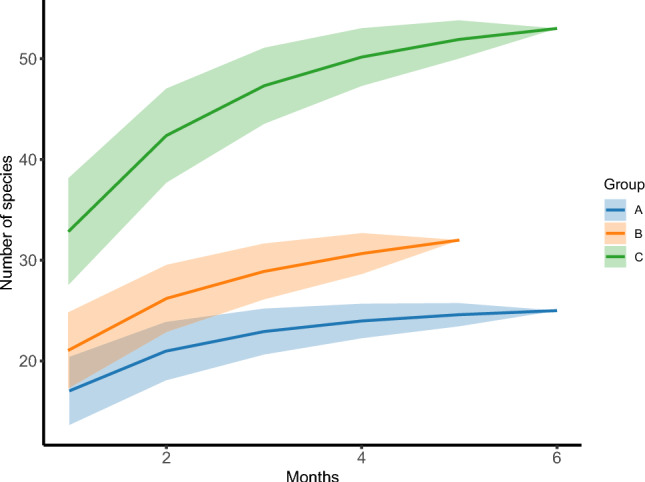
Richness of fruit species consumed by reintroduced woolly monkeys (Groups A, B, and C) at Reserve Rey Zamuro–Matarredonda: rarefaction curves with 95% confidence intervals. The x-axis represents the sampling effort or the number of monitoring months, while y-axis represents the cumulative species richness. Each curve corresponds to one of three groups: Group A (blue), Group B (orange), Group C (green), and the 95% confidence intervals are depicted as shaded regions around the curves. In this figure, we can observe how the accumulation of fruit species varies across the groups

Regarding fruit species, Group A consumed 25 plant species from 16 families, Group B 31 plant species from 14 families, and Group C 53 species from 24 families (Fig. 2). Notably, all three groups tended to reach an asymptote, indicating that the sampling effort was sufficient to estimate the richness of fruit species in the diets of the three groups.

The different fruit species consumed by the three groups based on the number of feeding bouts are reported on Supplementary information. For Group A the most important species were *Couma macrocarpa, Sarcaulus brasiliensis* and *Bellucia grossularioides*. For Group B, *Diospyros pseudoxylopia, Oenocarpus mapora,* and *Protium hepatphyllum.* For Group C, *Sarcaulus brasiliensis, Diospyros pseudoxylopia*, *Couma macrocarpa, Socratea exhorriza,* and *Virola elongata.* In many cases, the groups revisited individual trees of the mentioned and other species in the same and in different years.

## Use of space

### Home range

**Fig. 3:**
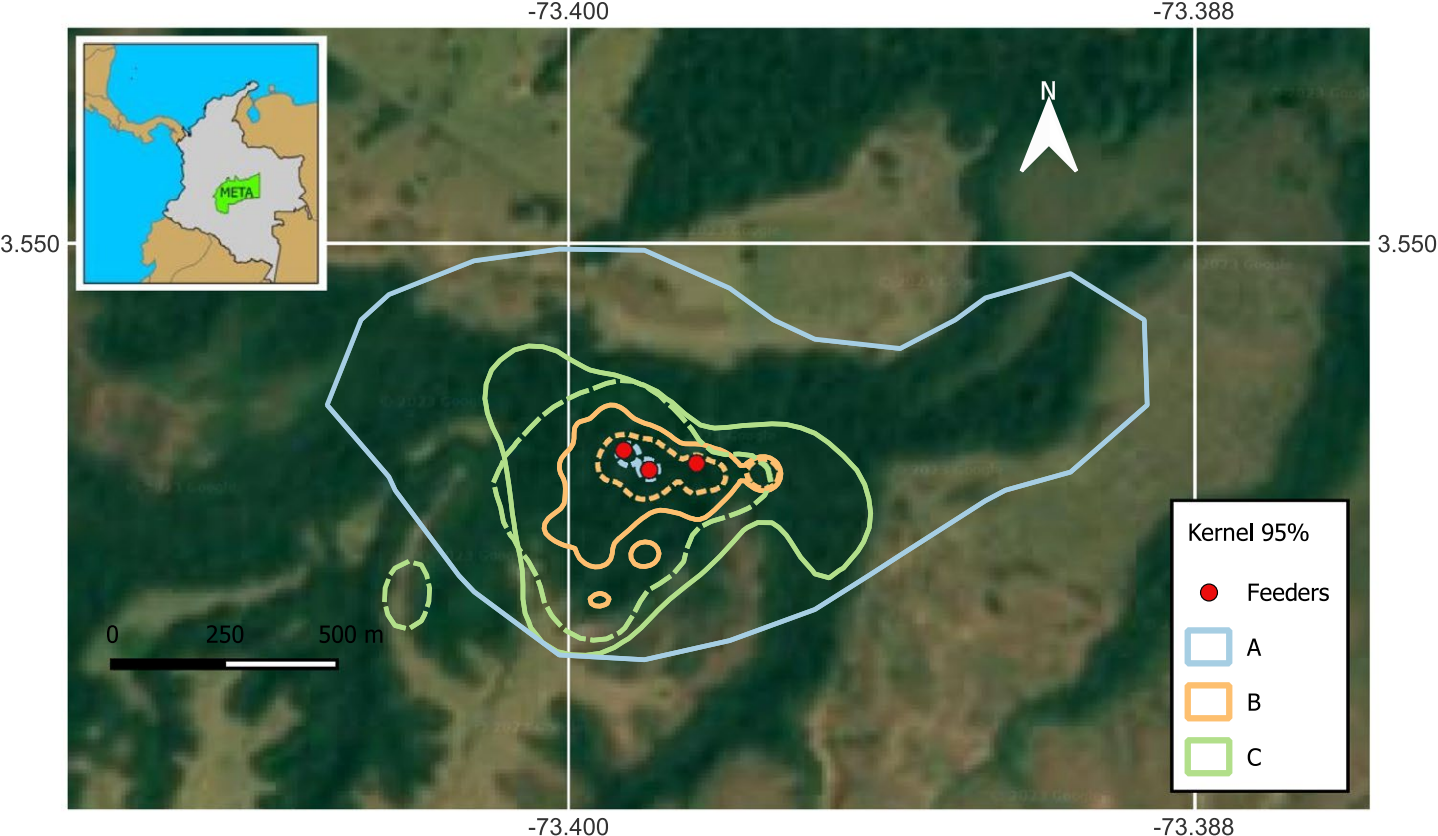
Home range estimation for the three groups of reintroduced woolly monkeys in Reserve Rey Zamuro–Matarredonda using kernel 95% analysis. Groups are represented by different colors: Group A (blue), Group B (orange), and Group C (green). The dotted lines represent the estimated home range for the first 2 months, while the continuous line depicts the home range for 6 months. The map shows the department of Colombia where the study area is located and the location of the feeders

According to kernel densities, at 2 months, the 95% home range was 0.34 ha for Group A, 2.97 ha for Group B, and 21.67 ha for Group C. These values increased to 107.95 ha, 9.42 ha, and 33.1 ha at 6 months for the same groups, respectively (Fig. 3). The 50% core areas were smaller and not necessarily concentrated in the same locations (Supplementary information). Particularly, Group A remained predominantly in the feeding areas. Rarefaction curves showed the same patterns, although area values were lower compared to kernel densities. Also, the rarefaction curves of none of the groups reached an asymptote (Supplementary information).

### Vertical strata

**Fig. 4:**
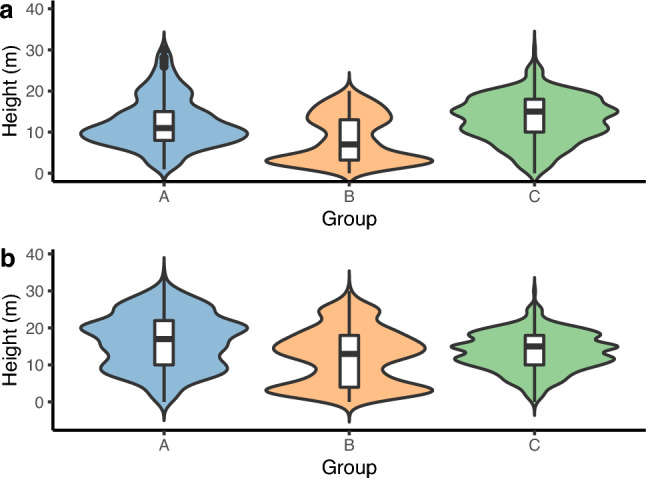
Violin plots of the estimated height above the ground of reintroduced woolly monkeys (Groups A, B and C) in Reserve Rey Zamuro–Matarredonda for the a) first 2 months after the release and b) total period of monitoring. Each violin plot provides a visual representation of the data distribution through the width of the violin. Box plots illustrate the upper and lower quartiles, with the median indicated by a line. The whiskers extend to show the highest and lowest values, while excluding outliers that are denoted by dots

For the first 2 months (Fig. 4a), Group C preferred higher vertical strata, while Group B used lower strata (KW: H = 137.48, df = 2, *p* < 0.001; Dunn’s post hoc test, *p* < 0.05). For the 6-month period (Fig. 4b), Group A used higher strata, and Group B occupied lower strata (KW: H = 150.7, df = 2, *p* < 0.001; Dunn’s post hoc test, *p* < 0.05). No other comparison between groups was significant (*p* > 0.05). However, it is worth noting that analyzing the 6-month period, the differences between the groups were less pronounced.

## Discussion

Our results suggest a positive influence of the presence of experienced conspecifics in the behavioral adaptation of reintroduced woolly monkeys, particularly during the initial 2 months. This rapid adaptation plays a crucial role in improving the chances of survival for reintroduced individuals (Whitehead 2010). Furthermore, diet and space utilization patterns tended to homogenize among the three groups over the entire monitoring period.

However, individuals from Group B had a lower percentage of proximity with experienced conspecifics than Group C (Table 2, Supplementary information), which could have resulted in fewer learning opportunities. Despite this, their association rates were similar to those observed in wild populations, with males showing higher association rates than females (Stevenson et al. 2015).

Regarding diet composition, our results indicated that, during the initial 2 months, Group A, without the presence of experienced conspecifics, had a different diet compared to Groups B and C. In contrast, Groups B and C, with access to experienced individuals shared less dissimilar diets in this phase. For instance, the diet of Group A was mainly based on the provided food, which is consistent with our prediction. Moreover, newly released individuals often consumed plants that are never seen in the diet of wild woolly monkeys (e.g., leaves of ferns).

It is worth noting that Group C exhibited the most diverse diet, including a greater number of fruit species that are commonly consumed by wild woolly monkeys, followed by Group B and then Group A. This trend may be associated with the fact that experienced individuals played a crucial role in locating these food sources. Although the three groups were released in different years, they all experienced the same season. Assuming that seasonal forest patterns tend to remain consistent across years (e.g., Stevenson 2004a; Stevenson et al. 2008), differences in the diet composition among reintroduced woolly monkey groups may be attributed to variations in their patterns of spatial utilization and experience. This is because a diet comprising a wider variety of fruits often requires the exploration of larger areas, as fruit resources can be unevenly distributed in terms of both space and time (Lim et al. 2021). Consequently, Groups B and C likely benefited from the knowledge of the experienced individuals. Many primate species have the ability to remember the locations of their food sources and may use mental maps to plan various foraging strategies (Trapanese et al. 2019). For instance, Russon (2002) found that newly reintroduced orangutans only began consuming forest food after observing other orangutans doing so, suggesting that the recognition of novel food sources may be linked to social learning. Locating fruits is generally more challenging than finding leaves; therefore, consuming a broader variety of fruits require enhanced skills, with social interactions playing a significant role, especially for fruits that are irregularly accessible in terms of timing and location (Russon 2008). For example, during focal samplings, we frequently observed newly reintroduced individuals to feed only after the experienced individuals had already located feeding trees and started feeding. According to the above, having a more diverse diet can be beneficial for individuals, particularly considering the variable availability of fruit species. This adaptability allows them to adjust or supplement their core diet as needed (Russon 2002).

In primate reintroduction programs, foraging inefficiency has been reported as one of the main challenges for individuals (Stoinski et al. 2003). Therefore, it is important to explore factors influencing the diet of reintroduced woolly monkeys. The practice of food provisioning is recommended to assist individuals in establishing in a specific location (Baker 2002) and to ensure they meet their caloric requirements while becoming familiar with wild foods. However, it predominantly consisted of cultivated foods, which do not necessarily align with their nutritional requirements (Milton 1999). Notably, research conducted in captivity has shown that this mismatch can affect their behavior (Guzmán-Caro and Stevenson 2014). Therefore, for optimal dietary balance and food source recognition, especially during the *in-situ* acclimatization phase, we recommend incorporating wild fruits for the individuals.

On the contrary, Groups B and C relied more on wild fruits and leaves, which align more closely with the natural diets of woolly monkeys in the wild. This natural diet includes fruits, but they also feed on leaves, arthropods, flowers, seeds, and small vertebrates (Defler and Defler 1996; González and Stevenson 2010; Stevenson et al. 1994). Woolly monkeys tend to consume a wider variety of resources when fruits are scarce (Stevenson et al. 1994). This dietary diversity is crucial for nutrient balancing, which is one of the nutritional goals of wild primates (Felton et al. 2009a). Additionally, a shift toward a more natural diet can positively influence the diversity of gut microbiota, potentially benefiting the fitness of these individuals (Quiroga-González et al. 2021). Therefore, it is crucial for reintroduced woolly monkeys to learn to utilize a wider range of non-toxic available resources.

In the context of fruit species, research conducted by Stevenson (2004b) found that woolly monkeys’ fruit preferences are influenced by factors such as abundance and astringency, which is a proxy for secondary compounds. For instance, species like *Oenocarpus mapora* and *Oenocarpus bataua*, are typically considered unpalatable and are not consumed by wild woolly monkeys (Stevenson 2004b). However, it is interesting to note that these species were present in the diet of Group B, but then their consumption decreased for group C. We speculate that the inclusion of these plants could be generated from observing other primate species such as *Sapajus apella* and *Alouatta seniculus*, as they are common dietary sources for these species (Stevenson et al. 2000a, b), but perhaps digestive problems leaded to its rejection.

In these groups, woolly monkeys may have relied on the knowledge and behaviors of their experienced conspecifics to identify and incorporate a wider range of wild fruits and natural food sources. However, over the total monitoring period, the differences in diet composition between Group A and the other groups diminished, with all three groups exhibiting more similar diets. This convergence in diet composition could be attributed to a learning curve that extended beyond the initial 2 months. As Group A continued to adapt to its environment, individuals might have progressively incorporated wild food sources and broadened their diet repertoire. Additionally, the gradual reduction in the frequency of human food supply likely encouraged all groups to rely more on foraging and explore a wider range of food resources.

These results align with the idea that social learning plays a significant role in diet composition (Chapman and Fedigan 1990; Gunst et al. 2008; Snowdon and Boe 2003), ultimately positively influencing the adaptation of reintroduced woolly monkey individuals to their new environment.

Regarding home range, for the first 2 months, results indicated that Group C had the largest home range, followed by Groups B and A, being consistent with the formulated prediction. This pattern suggests that the presence of experienced conspecifics might have accelerated the exploration and expansion of the home range by Groups B and C. Notably, newly reintroduced individuals were frequently observed following more experienced ones, facilitating their exploration of the environment, which is consistent with the *copy-successful-individuals* learning strategy (Laland 2004). Woolly monkeys, being group-living animals and considered as cohesive in most lowland populations, tend to move guided by social cues and interactions with other group members (Ellis and Di Fiore 2019; Stevenson 1998). Proximity to conspecifics has been considered as one of the principal components to evaluate movement patterns and, consequently, reintroduction success, driven by conspecific attraction (Berger-Tal and Saltz 2014; Le Gouar et al. 2012). This attraction leads to the clustering of individuals in specific locations (Gil et al. 2018). According to Stamps (1988), conspecific attraction and territorial clustering might occur for four reasons: mating success, predator protection, defense against competitors, and information about the habitat.

However, over the total monitoring period, the home range areas of all groups expanded, with Group A eventually having the largest home range area, followed by Group C and Group B.

Notably, rarefaction curves of none of the groups reached an asymptote, suggesting their use of larger areas. This expansion could be attributed to a potential trade-off between exploratory and exploitative behaviors (Berger-Tal and Saltz 2014). Notably, Group A, which lacked the advantages of social learning, displayed movement patterns that leaned more towards exploration rather than staying in one place for exploitation. Furthermore, given that at the end of the monitoring period, Group A only had one survivor, Micaela, this variation in home range size might also be attributed to individual differences. Temperament, defined as individual characteristics influencing and regulating behavior, can significantly impact success of reintroduction programs (McDougall et al. 2006; Watters and Meehan 2007). For instance, some individuals might be more inclined to explore (Berger-Tal and Saltz 2014). Over time, despite interacting with other group members, Micaela consistently displayed greater movement patterns compared to the other individuals. We believe that Micaela had previous experience in natural forests, because right after her release, she agilely climbed to the forest canopy, where she engaged in various behaviors without any kind of accident.

For primates, home range size may vary due to different ecological processes or group size (Defler 1996; González and Stevenson 2010; Stevenson 2006; Stevenson et al. 1994). However, considering that wild woolly monkeys live in groups between 10 and 45 individuals (Defler 1996; Kavanagh and Dresdale 1975; Stevenson 1998) and that all groups in the study had a maximum of seven individuals, group size may not have significantly affected home range size. Regardless of size, the establishment of a home range is an indicator of behavioral adaptation for reintroduced individuals. In fact, two individuals (adult males from Groups B and C) were lost after release due to high post-release dispersal and falls to the ground, impeding their establishment. For the remaining individuals, an important factor of the home range establishment might have been the presence of feeding platforms and acclimatization enclosures, as familiarity with habitat cues can impact the preference of an individual for settling in that habitat (Stamps and Swaisgood 2007). Indeed, core areas coincided with location of feeding platforms, even during time periods when there was no provisioning. This might have limited the natural movements of reintroduced individuals, creating a small home range induced by the activities of human observers (Millán et al. 2014). However, as previously mentioned, this practice is recommended for initial establishment of individuals. Consequently, it is important for further studies to evaluate how to address this to improve the use of space by reintroduced individuals.

Regarding vertical strata usage, significant differences were observed among all three groups during both the first 2 months and the total monitoring period. Group C, with the advantage of individuals familiar with the environment, utilized higher vertical strata faster compared to Groups A and B. This suggests that the presence of experienced conspecifics might have influenced the use of higher strata within the canopy (Schwartz et al. 2016). However, as in home range, it is important to consider interindividual variations. This may explain why Group A showed a faster utilization of higher vertical strata than Group B. Notably, Micaela consistently avoided descending to the ground after release, in contrast to other individuals of the same Group (Supplementary Information).

The presence of feeding platforms also may have influenced vertical strata usage. While these platforms were placed in trees to stimulate monkeys to ascend, fruits and vegetables frequently fell to the ground during feeding bouts, inducing individuals to descend to obtain the remaining food. This behavior was particularly noticeable only during the initial months. For example, most of the instances when individuals from Group B were recorded at low altitudes were associated with feeding events involving the consumption of remaining food. Moreover, when Group C began relying on wild fruits, there were days when they did not visit the feeding platforms, leaving fruits uneaten and consequently reducing the number of events of descending to the ground.

The use of vertical strata might not be solely related to ecological factors. For example, research on wild muriqui monkeys (*Brachyteles hypoxanthus*) showed that one group developed a terrestrial tradition, likely through social learning process (Tabacow et al. 2009). In contrast, woolly monkeys prefer the middle and lower portions of the canopy (Defler 1999). This behavior serves as a defense strategy against both aerial predators, such as eagles, and terrestrial predators, like felids (Schmitt and Di Fiore 2014). The utilization of high vertical strata is crucial for reintroduced woolly monkeys, as they must learn to avoid predators, and anti-predator behavior has been reported as one of the challenges in reintroduction programs (Berger-Tal et al. 2020).

### Implications for conservation

One of the main reasons for the failure of reintroduction efforts often stems from a lack of understanding of the specific requirements of the species being reintroduced (Cheyne et al. 2012). In the case of cohesive social animals like woolly monkeys, social interactions are important. Recognizing this vital aspect is not only crucial for the development of successful reintroduction programs (Konstant and Mittermeier 1982), but also for the effective management and rehabilitation of captive primates. This study offers relevant insights for future neotropical primate rehabilitation and reintroduction programs, particularly due to limited data available on these processes. Also, it is important to consider that the extent of learning and its impact on fitness can vary based on species-specific life history traits and individual characteristics (Rochais et al. 2022).

**Fig. 5:**
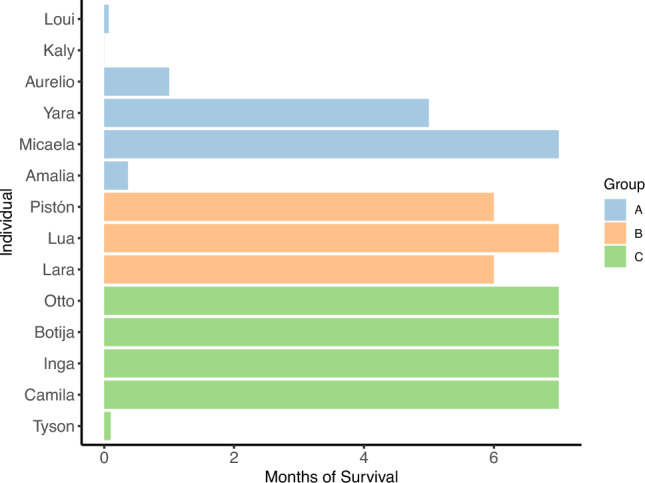
Survival of reintroduced woolly monkeys in Reserve Rey Zamuro–Matarredonda. This bar chart presents survival data for reintroduced woolly monkeys to the Reserve Rey Zamuro–Matarredonda during their initial 6 months post-release. The x-axis tracks the “Months of Survival”, while the y-axis indicates the “Individual”. Each color corresponds to one of the three groups: Group A (blue), Group B (orange) and Group C (green). Graphs were generated using the ggplot2 package (Wickham 2016) in R version 4.1.1 (R Core Team 2021)

The presence of experienced conspecifics played a critical role in the rapid behavioral adaptation of reintroduced woolly monkeys, particularly in terms of diet diversity and use of space. This could be evidenced in the increased survival of individuals from the groups with access to experienced conspecifics during the first 7 months following reintroduction (Fig. 5), as Group A. with no access, lost four individuals in the 1st month.

These findings suggest that incorporating individuals with prior wild experience into reintroduction groups can significantly enhance the chances of success in reintroduction programs for woolly monkeys and, potentially, for other primate species as well. Moreover, as observed in this case, reinforcements of previously released groups may also underscore the advantages of social learning. Conservation efforts aiming to reintroduce primates back into their natural habitats should consider the benefits of social learning throughout the process. In this context, the use of soft releases involving acclimatization enclosures not only aids in the adaptation of new individuals to their environment but also facilitates interaction between established and newly introduced group members, promoting group cohesion post-release.

Furthermore, the gradual convergence in diet composition among reintroduced groups over time highlights the potential for individuals to adapt and learn from their environment, even in the absence of experienced conspecifics, although this process may extend beyond the initial phase.

Regarding management in captivity, practices that promote social processes can be crucial (Hopper 2021). The opportunities for social learning can vary based on the specific social context. While woolly monkey relationships are typically characterized as tolerant (Di Fiore and Fleischer 2005), instances of social conflicts among group members may still arise. However, proactive measures can be taken to create an environment conductive to these processes (Schwartz et al. 2016), considering the different behavioral types present in the group (Watters and Meehan 2007). For other primate species, the existing hierarchy within groups should be carefully considered when facilitating social learning opportunities.

In conclusion, this study highlights the important role of the presence of experienced conspecifics and social learning in the initial adaptation of reintroduced woolly monkeys. These findings have important implications for primate conservation efforts, emphasizing the need to include individuals with prior wild experience in reintroduction programs.

## Supplementary Information

Below is the link to the electronic supplementary material.

Supplementary file1 (DOCX 18 KB)

Supplementary file2 (PDF 315 KB)

Supplementary file3 (DOCX 14 KB)

Supplementary file4 (PDF 5 KB)

Supplementary file5 (DOCX 14 KB)

Supplementary file6 (PDF 5 KB)

Supplementary file7 (DOCX 15 KB)

Supplementary file8 (PDF 42 KB)

Supplementary file9 (DOCX 14 KB)

Supplementary file10 (PDF 23 KB)

Supplementary file11 (DOCX 14 KB)

Supplementary file12 (PDF 27 KB)

Supplementary file13 (DOCX 23 KB)
