# Supplementary figures and images for: The presence of experienced individuals enhance the behavior and survival of reintroduced woolly monkeys in Colombia
Source: Primates. 2024 Oct 25;66(1):103–15. doi: 10.1007/s10329-024-01156-2 (PMC11735561; doi:10.1007/s10329-024-01156-2)

3.550

-73.400

-73.388

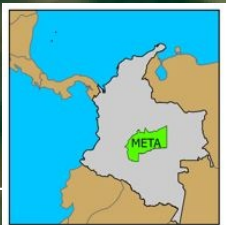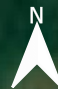

3.550

0 250 500 m

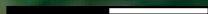

-73.400

-73.388

Kernel 50%

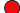 Feeders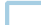 A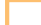 B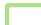 C

Supplement: Supplementary file 2 — Supplementary file2 (PDF 315 KB) [file 10329_2024_1156_MOESM2_ESM.pdf]

**a**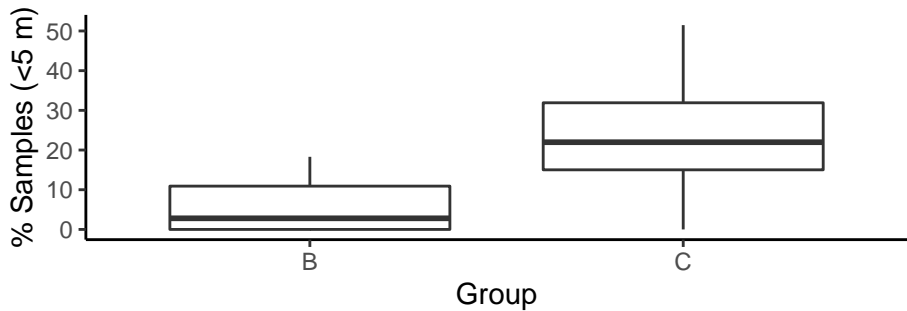**b**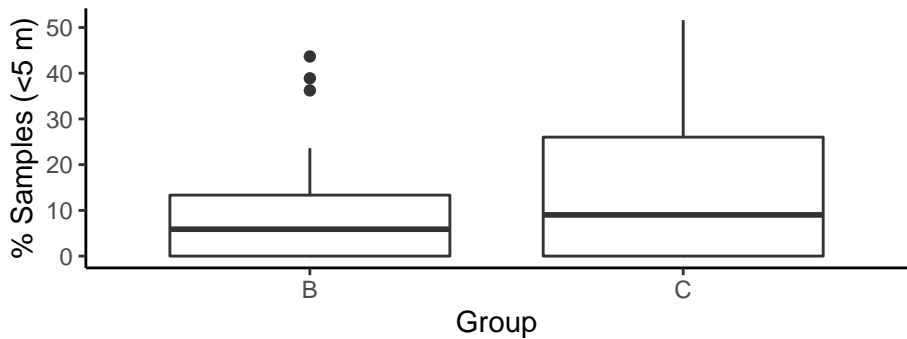

Supplement: Supplementary file 4 — Supplementary file4 (PDF 5 KB) [file 10329_2024_1156_MOESM4_ESM.pdf]

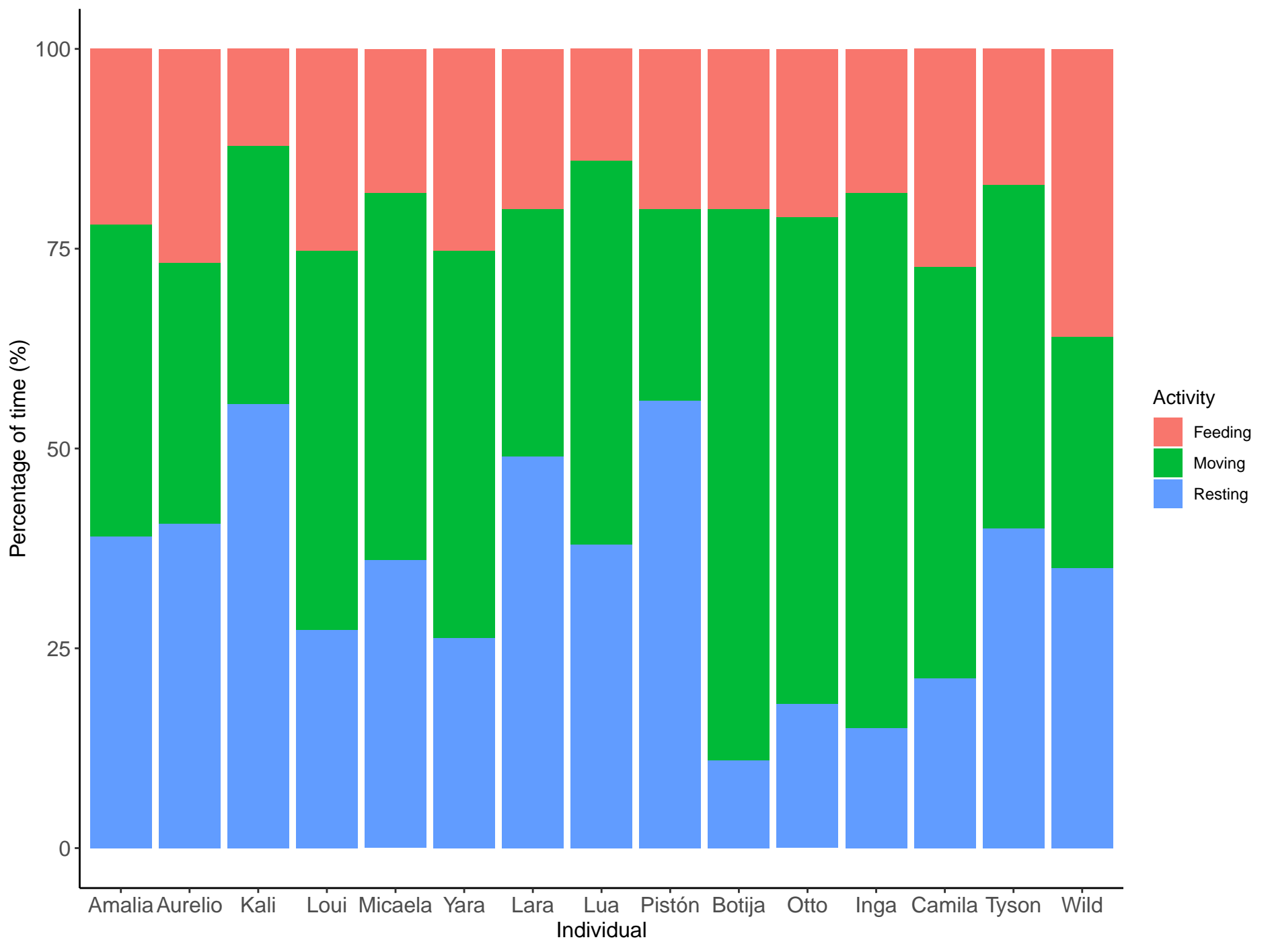

Supplement: Supplementary file 6 — Supplementary file6 (PDF 5 KB) [file 10329_2024_1156_MOESM6_ESM.pdf]

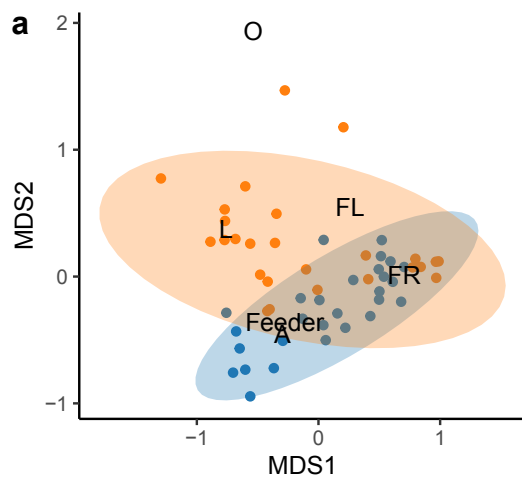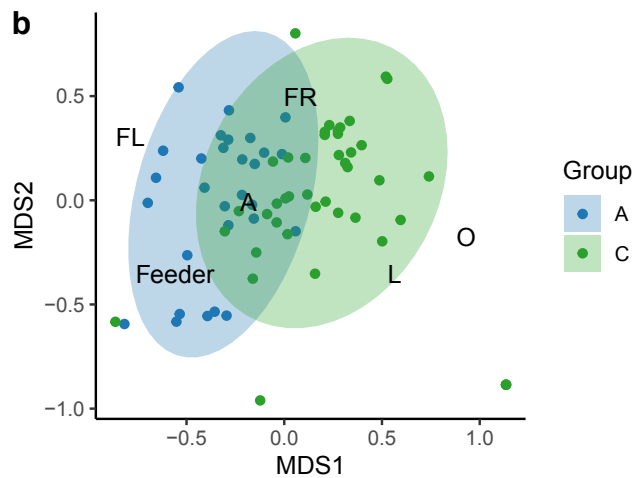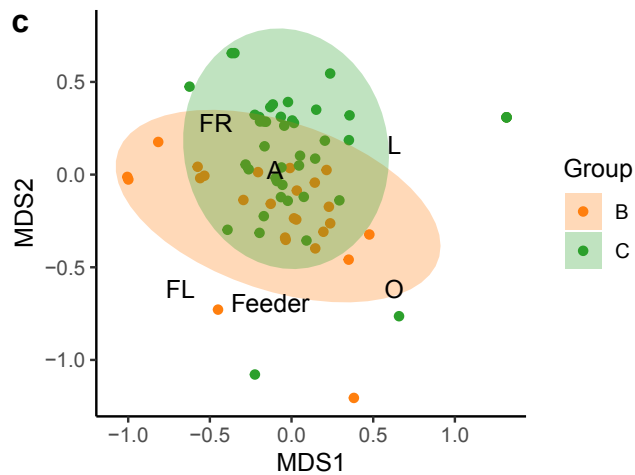

Supplement: Supplementary file 8 — Supplementary file8 (PDF 42 KB) [file 10329_2024_1156_MOESM8_ESM.pdf]

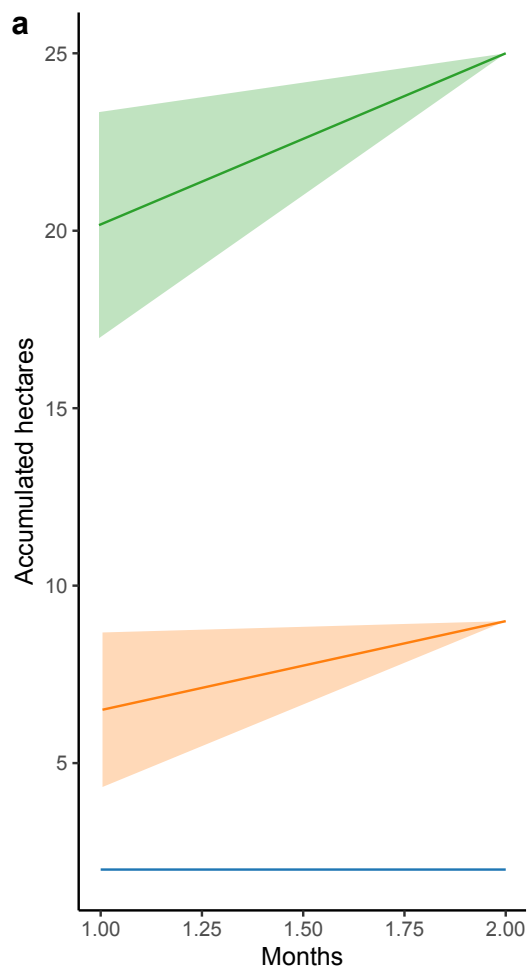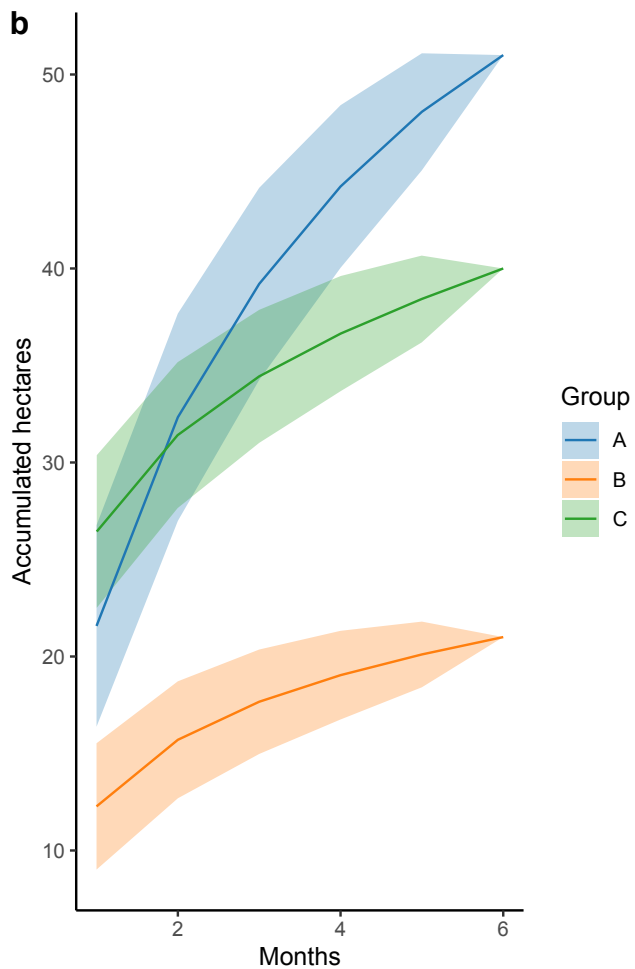

Supplement: Supplementary file 10 — Supplementary file10 (PDF 23 KB) [file 10329_2024_1156_MOESM10_ESM.pdf]

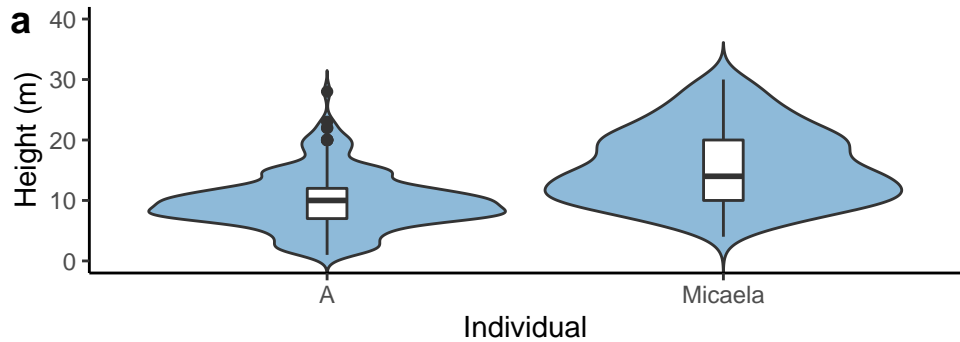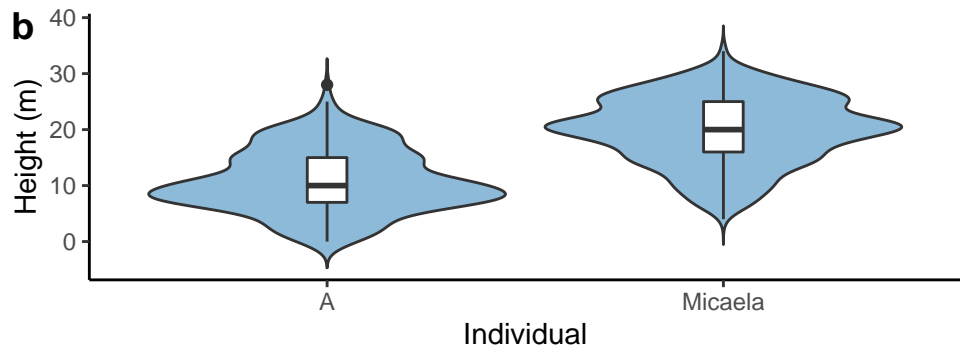

Supplement: Supplementary file 12 — Supplementary file12 (PDF 27 KB) [file 10329_2024_1156_MOESM12_ESM.pdf]
