# Supplementary material for: The presence of experienced individuals enhance the behavior and survival of reintroduced woolly monkeys in Colombia
Source: Primates. 2024 Oct 25;66(1):103–15. doi: 10.1007/s10329-024-01156-2 (PMC11735561; doi:10.1007/s10329-024-01156-2)
Supplement: Supplementary file 3 — Supplementary file3 (DOCX 14 KB) [file 10329_2024_1156_MOESM3_ESM.docx]

# **The presence of experienced individuals enhance the behavior and survival of reintroduced woolly monkeys in Colombia.**

**Journal:** Primates

Mariana Gómez-Muñoz^1^, Mónica A. Ramírez^2^, Jairo Pérez-Torres^3^ and Pablo R. Stevenson^2^

^1^Facultad de Estudios Ambientales y Rurales, Pontificia Universidad Javeriana, Bogotá, Colombia, ^2^Laboratorio de Ecología de Bosques Tropicales y Primatología (LEBTYP), Departamento de Ciencias Biológicas, Universidad de Los Andes, Bogotá, Colombia., ^3^Laboratorio de Ecología Funcional (LEF), Unidad de Ecología y Sistemática (UNESIS), Departamento de Biología, Facultad de Ciencias, Pontificia Universidad Javeriana, Bogotá, Colombia

**Corresponding author:** Mariana Gómez-Muñoz, Email: mariana.gomezm@javeriana.edu.co

**Appendix 6** Percentage of instantaneous samples in which focal animals of Groups B and C were in less than 5 m from conspecifics (including experienced individuals) for a) first two months, b) six months. In three wild ranging populations of woolly monkeys mean percentage of samples vary between 10 and 40 (Stevenson et al. 2015), suggesting that our reintroduced woolly monkeys behave at least as cohesive as natural populations.
