# Supplementary material for: The presence of experienced individuals enhance the behavior and survival of reintroduced woolly monkeys in Colombia
Source: Primates. 2024 Oct 25;66(1):103–15. doi: 10.1007/s10329-024-01156-2 (PMC11735561; doi:10.1007/s10329-024-01156-2)
Supplement: Supplementary file 5 — Supplementary file5 (DOCX 14 KB) [file 10329_2024_1156_MOESM5_ESM.docx]

# **The presence of experienced individuals enhance the behavior and survival of reintroduced woolly monkeys in Colombia.**

**Journal:** Primates

Mariana Gómez-Muñoz^1^, Mónica A. Ramírez^2^, Jairo Pérez-Torres^3^ and Pablo R. Stevenson^2^

^1^Facultad de Estudios Ambientales y Rurales, Pontificia Universidad Javeriana, Bogotá, Colombia, ^2^Laboratorio de Ecología de Bosques Tropicales y Primatología (LEBTYP), Departamento de Ciencias Biológicas, Universidad de Los Andes, Bogotá, Colombia., ^3^Laboratorio de Ecología Funcional (LEF), Unidad de Ecología y Sistemática (UNESIS), Departamento de Biología, Facultad de Ciencias, Pontificia Universidad Javeriana, Bogotá, Colombia

**Corresponding author:** Mariana Gómez-Muñoz, Email: mariana.gomezm@javeriana.edu.co

**Appendix 1** Percentage of time spent by reintroduced and wild woolly monkeys on different activities (Feeding, Moving and Resting). X2 values can be found in Appendix 2.
