# Supplementary material for: The presence of experienced individuals enhance the behavior and survival of reintroduced woolly monkeys in Colombia
Source: Primates. 2024 Oct 25;66(1):103–15. doi: 10.1007/s10329-024-01156-2 (PMC11735561; doi:10.1007/s10329-024-01156-2)
Supplement: Supplementary file 7 — Supplementary file7 (DOCX 15 KB) [file 10329_2024_1156_MOESM7_ESM.docx]

# **The presence of experienced individuals enhance the behavior and survival of reintroduced woolly monkeys in Colombia.**

**Journal:** Primates

Mariana Gómez-Muñoz^1^, Mónica A. Ramírez^2^, Jairo Pérez-Torres^3^ and Pablo R. Stevenson^2^

^1^Facultad de Estudios Ambientales y Rurales, Pontificia Universidad Javeriana, Bogotá, Colombia, ^2^Laboratorio de Ecología de Bosques Tropicales y Primatología (LEBTYP), Departamento de Ciencias Biológicas, Universidad de Los Andes, Bogotá, Colombia., ^3^Laboratorio de Ecología Funcional (LEF), Unidad de Ecología y Sistemática (UNESIS), Departamento de Biología, Facultad de Ciencias, Pontificia Universidad Javeriana, Bogotá, Colombia

**Corresponding author:** Mariana Gómez-Muñoz, Email: mariana.gomezm@javeriana.edu.co

**Appendix 2** Diet composition variation among reintroduced woolly monkeys (Groups A, B and C) at Reserve Rey Zamuro. Non-metric multidimensional scaling ordination (NMDS) of diet composition during a) the total monitoring period of Groups A and B, b) A and C and c) B and C. The diet components considered include Arthropods (A), Fruits (FR), Flowers (FL), Feeder, Leaves (L) and Others (O). The x-axis represents NMDS Axis 1, and the y-axis represents NMDS Axis 2. In the plot, different groups (A, B and C) are distinguished by color, with Group A represented by blue ellipse, Group B by orange ellipse and Group C by green ellipse. This analysis visually shows dietary variations among the three groups.

Diet composition obtained from NMDS showed a similar composition between Groups A and B (stress = 0.125) (a), between A and C (stress = 0.147) (b) and between B and C (0.142) (c). The ANOSIM analysis also showed dissimilarity in diet composition between A and B (R = 0.10, p = 0.011), between A and C (R = 0.20, p = 0.001) but no statistical significance difference between B and C (R = 0.04, p = 0.137)
