# Supplementary material for: The presence of experienced individuals enhance the behavior and survival of reintroduced woolly monkeys in Colombia
Source: Primates. 2024 Oct 25;66(1):103–15. doi: 10.1007/s10329-024-01156-2 (PMC11735561; doi:10.1007/s10329-024-01156-2)
Supplement: Supplementary file 9 — Supplementary file9 (DOCX 14 KB) [file 10329_2024_1156_MOESM9_ESM.docx]

# **The presence of experienced individuals enhance the behavior and survival of reintroduced woolly monkeys in Colombia.**

**Journal:** Primates

Mariana Gómez-Muñoz^1^, Mónica A. Ramírez^2^, Jairo Pérez-Torres^3^ and Pablo R. Stevenson^2^

^1^Facultad de Estudios Ambientales y Rurales, Pontificia Universidad Javeriana, Bogotá, Colombia, ^2^Laboratorio de Ecología de Bosques Tropicales y Primatología (LEBTYP), Departamento de Ciencias Biológicas, Universidad de Los Andes, Bogotá, Colombia., ^3^Laboratorio de Ecología Funcional (LEF), Unidad de Ecología y Sistemática (UNESIS), Departamento de Biología, Facultad de Ciencias, Pontificia Universidad Javeriana, Bogotá, Colombia

**Corresponding author:** Mariana Gómez-Muñoz, Email: mariana.gomezm@javeriana.edu.co

**Appendix 5** Rarefaction curves representing the frequency of visits to each quadrant by each group of reintroduced woolly monkeys in Reserve Rey Zamuro-Matarredonda as a function of time (months). Panel a) focuses on the first two months of monitoring, while panel b) encompasses the total monitoring period. The x-axis in both panels represents the time in months, reflecting sampling effort, while the y-axis represents accumulated hectares as the frequency of visits to specific 1 ha quadrants within the study area. Each curve corresponds to one of the three groups: Group A (blue), Group B (orange) and Group C (green).
