# Supplementary material for: The presence of experienced individuals enhance the behavior and survival of reintroduced woolly monkeys in Colombia
Source: Primates. 2024 Oct 25;66(1):103–15. doi: 10.1007/s10329-024-01156-2 (PMC11735561; doi:10.1007/s10329-024-01156-2)
Supplement: Supplementary file 11 — Supplementary file11 (DOCX 14 KB) [file 10329_2024_1156_MOESM11_ESM.docx]

# **The presence of experienced individuals enhance the behavior and survival of reintroduced woolly monkeys in Colombia.**

**Journal:** Primates

Mariana Gómez-Muñoz^1^, Mónica A. Ramírez^2^, Jairo Pérez-Torres^3^ and Pablo R. Stevenson^2^

^1^Facultad de Estudios Ambientales y Rurales, Pontificia Universidad Javeriana, Bogotá, Colombia, ^2^Laboratorio de Ecología de Bosques Tropicales y Primatología (LEBTYP), Departamento de Ciencias Biológicas, Universidad de Los Andes, Bogotá, Colombia., ^3^Laboratorio de Ecología Funcional (LEF), Unidad de Ecología y Sistemática (UNESIS), Departamento de Biología, Facultad de Ciencias, Pontificia Universidad Javeriana, Bogotá, Colombia

**Corresponding author:** Mariana Gómez-Muñoz, Email: mariana.gomezm@javeriana.edu.co

**Appendix 7** Violin plots of the estimated height above the ground of reintroduced woolly monkeys, specially focusing on Micaela and the other individuals in Group A in Reserve Rey Zamuro-Matarredonda for the a) first two months after the release and b) total period of monitoring. Each violin plot provides a visual representation of the data distribution through the width of the violin. Box plots illustrate the upper and lower quartiles, with the median indicated by a line. The whiskers extend to show the highest and lowest values, while excluding outliers that are denoted by dots.
