# Supplementary material for: The presence of experienced individuals enhance the behavior and survival of reintroduced woolly monkeys in Colombia
Source: Primates. 2024 Oct 25;66(1):103–15. doi: 10.1007/s10329-024-01156-2 (PMC11735561; doi:10.1007/s10329-024-01156-2)
Supplement: Supplementary file 13 — Supplementary file13 (DOCX 23 KB) [file 10329_2024_1156_MOESM13_ESM.docx]

# **The presence of experienced individuals enhance the behavior and survival of reintroduced woolly monkeys in Colombia.**

**Journal:** Primates

Mariana Gómez-Muñoz^1^, Mónica A. Ramírez^2^, Jairo Pérez-Torres^3^ and Pablo R. Stevenson^2^

^1^Facultad de Estudios Ambientales y Rurales, Pontificia Universidad Javeriana, Bogotá, Colombia, ^2^Laboratorio de Ecología de Bosques Tropicales y Primatología (LEBTYP), Departamento de Ciencias Biológicas, Universidad de Los Andes, Bogotá, Colombia., ^3^Laboratorio de Ecología Funcional (LEF), Unidad de Ecología y Sistemática (UNESIS), Departamento de Biología, Facultad de Ciencias, Pontificia Universidad Javeriana, Bogotá, Colombia

**Corresponding author:** Mariana Gómez-Muñoz, Email: mariana.gomezm@javeriana.edu.co

**Appendix 3** Fruit species and corresponding families consumed by reintroduced woolly monkeys (Groups A, B and C) in Reserve Rey Zamuro based on number of feeding bouts

| **No. of feeding bouts per group** | | | **Species** | **Family** |
| --- | --- | --- | --- | --- |
| **A** | **B** | **C** |  |  |
| 39 | 2 | 175 | *Sarcaulus brasiliensis* | Sapotaceae |
| 0 | 66 | 116 | *Diospyros pseudoxylopia* | Ebenaceae |
| 43 | 0 | 63 | *Couma macrocarpa* | Apocynaceae |
| 5 | 4 | 47 | *Socratea exhorriza* | Arecaceae |
| 6 | 3 | 47 | *Virola elongata* | Myristicaceae |
| 0 | 0 | 39 | *Pseudolmedia laevis* | Moraceae |
| 0 | 5 | 38 | *Pleonotoma* sp. | Bignoniaceae |
| 13 | 22 | 37 | *Protium heptaphyllum* | Burseraceae |
| 0 | 0 | 34 | *Miconia trinervia* | Melastomataceae |
| 19 | 7 | 31 | *Pseudolmedia hirsuta* | Moraceae |
| 9 | 9 | 28 | *Hymenaea oblongifolia* | Fabaceae |
| 5 | 13 | 21 | *Hieronyma alchorneoides* | Phyllantaceae |
| 30 | 19 | 15 | *Bellucia grossularioides* | Melastomataceae |
| 0 | 0 | 13 | *Orthomene schomburgkii* | Menispermaceae |
| 0 | 0 | 11 | *Ocotea floribunda* | Lauraceae |
| 0 | 9 | 8 | *Cecropia sciadophylla* | Urticaceae |
| 0 | 0 | 8 | *Alchorneopsis floribunda* | Euphorbiaceae |
| 0 | 0 | 8 | *Licania kunthiana* | Chrysoblanaceae |
| 0 | 0 | 8 | *Maripa panamensis* | Convolvulaceae |
| 0 | 1 | 7 | *Protium rhoifolium* | Burseraceae |
| 0 | 1 | 7 | *Trattinnickia rhoifolia* | Burseraceae |
| 3 | 21 | 6 | *Robrichia schomburgkii* | Fabaceae |
| 2 | 0 | 6 | *Ficus* sp. | Moraceae |
| 1 | 0 | 6 | *Paullinia ingifolia* | Sapindaceae |
| 0 | 0 | 6 | *Ficus cf. jimenezii* | Moraceae |
| 0 | 0 | 6 | *Norantea guianensis* | Marcgraviaceae |
| 0 | 15 | 5 | *Clarisia racemosa* | Moraceae |
| 10 | 0 | 5 | *Tapirira guianensis* | Anacardiaceae |
| 0 | 0 | 5 | *Anthurium gracile* | Araceae |
| 0 | 0 | 5 | *Inga thibaudiana* | Fabaceae |
| 0 | 0 | 5 | *Protium calanense* | Burseraceae |
| 0 | 5 | 4 | *Virola sebifera* | Myristicaceae |
| 0 | 1 | 4 | *Xylopia polyantha* | Annonaceae |
| 0 | 0 | 4 | *Protium llanorum* | Burseraceae |
| 0 | 48 | 3 | *Oenocarpus mapora* | Arecaceae |
| 21 | 0 | 3 | *Iryanthera laevis* | Myristicaceae |
| 0 | 20 | 3 | *Oenocarpus bataua* | Arecaceae |
| 0 | 0 | 3 | *Dialium guianense* | Fabaceae |
| 0 | 0 | 3 | *Ficus americana* | Moraceae |
| 0 | 0 | 3 | *Ficus dendrocida* | Moraceae |
| 0 | 11 | 2 | *Inga alba* | Fabaceae |
| 7 | 1 | 2 | *Guatteria punctata* | Annonaceae |
| 0 | 4 | 2 | *Ficus gomelleira* | Moraceae |
| 3 | 0 | 2 | *Ocotea longifolia* | Lauraceae |
| 0 | 0 | 2 | *Jupunba trapezifolia* | Fabaceae |
| 0 | 0 | 2 | *Brosimum guianense* | Moraceae |
| 0 | 0 | 2 | *Inga umbellifera* | Fabaceae |
| 0 | 0 | 2 | *Sloanea guianensis* | Elaeocarpaceae |
| 0 | 9 | 1 | *Aspidosperma spruceanum* | Apocynaceae |
| 5 | 0 | 1 | *Mauritia flexuosa* | Arecaceae |
| 0 | 4 | 1 | *Anemopaegma oligoneuron* | Bignoniaceae |
| 0 | 0 | 1 | *Cayaponia granatensis* | Cucurbitaceae |
| 0 | 0 | 1 | *Syagrus orinocensis* | Arecaceae |
| 29 | 0 | 0 | *Nectandra* sp*.* | Lauraceae |
| 28 | 0 | 0 | *Virola flexuosa* | Myristicaceae |
| 0 | 25 | 0 | *Parinari excelsa* | Chrysobalanaceae |
| 17 | 0 | 0 | *Goupia glabra* | Goupiaceae |
| 0 | 8 | 0 | *Adenocalymma cladotrichum* | Bignoniaceae |
| 5 | 0 | 0 | *Protium glabrescens* | Burseraceae |
| 0 | 5 | 0 | *Bactris sp.* | Arecaceae |
| 4 | 0 | 0 | *Perebea mollis* | Moraceae |
| 0 | 3 | 0 | *Pourouma minor* | Urticaceae |
| 3 | 0 | 0 | *Genipa americana* | Rubiaceae |
| 0 | 2 | 0 | *Fridericia patellifera* | Bignoniaceae |
| 0 | 2 | 0 | *Protium glabrescens* | Burseraceae |
| 2 | 0 | 0 | *Tovomita* sp. | Clusiaceae |
| 1 | 0 | 0 | *Euterpe precatoria* | Arecaceae |
| 0 | 1 | 0 | *Jacaranda* sp. | Bignoniaceae |
